# Human Motion Detection in Swimming Motion Video Based on Multiscale Separation Spatio‐Temporal Attention Mechanism

**DOI:** 10.1155/abb/3246852

**Published:** 2025-12-02

**Authors:** Jia Lu

**Affiliations:** ^1^ School of Physical Education, Zhengzhou University of Industrial Technology, Zhengzhou, 451150, China

**Keywords:** attention mechanisms, human motion detection, motion video, multiscale, swimming

## Abstract

Swimming motion video human motion detection is becoming increasingly important in sports training and event analysis. Existing methods are deficient in dealing with complex underwater environments and rapid changes in swimming movements, and the accuracy and real‐time performance of motion detection are low. Therefore, the study proposes a human motion detection method for swimming motion video based on multiscale (MS) separation spatio‐temporal attention mechanism (STAM). The encoder–decoder architecture extracts and fuses features of different scales in both spatial and temporal dimensions to realize automatic detection and precise localization of swimming motion. The experimental results indicated that the feature extraction accuracy reached 97.34% after 43 iterations, and the feature importance reached 0.982 after 40 iterations. In terms of recognition accuracy, the average accuracy of the model reached 94.02%, the recall rate was 93.09%, and the F1 score was 93.56%. Adaptive testing of movement changes showed that the detection accuracy generally remained above 89%, and the accuracy in slow and large‐sized movements even exceeded 95%. In addition to increasing swimming action detection’s precision and resilience, the work offers technological and theoretical backing for the creation of intelligent sports analysis systems.

## 1. Introduction

As computer vision and artificial intelligence technologies advance quickly, video analytics has emerged as a key area of study in a number of disciplines. Among them, human behavior recognition, especially in sports scenarios, has attracted much attention [1, 2]. Swimming, as a sport with high technical requirements and complex and varied movements, requires high precision of athletes’ movements. Therefore, the realization of intelligent detection of human movements in swimming sports videos has important research value [3, 4]. Through the intelligent analysis of swimming sports video, coaches and athletes can not only obtain real‐time technical evaluation but also provide scientific support for teaching, training, and event commentary. It can even provide data basis for the optimization of sports performance [5, 6]. Most current research focuses on simple action recognition based on RGB images or skeletal data. While these methods have achieved good results in static or single scenes, they perform poorly when facing complex scenes like swimming motion [7]. First, swimming movements take place in water, and complex background changes, such as water ripples and reflections, make video processing more difficult [8]. Second, the high speed and large gesture changes of swimming actions make it difficult for existing spatio‐temporal motion detection methods to accurately capture the time of occurrence and spatial location of the action [9]. Many methods struggle to effectively deal with dynamic changes in motion detection when dealing with uncropped long video sequences, while finding a balance between computational complexity and detection efficiency is difficult [10]. In addition, the multiscale (MS) elements in the video are frequently not fully utilized by current approaches, which reduces detection accuracy when dealing with delicate and quickly changing movements in swimming sports [11]. In view of this, the study proposes a human motion detection method for swimming motion video based on MS separation spatio‐temporal attention mechanism (STAM). The encoder–decoder architecture extracts and fuses features of different scales in both spatial and temporal dimensions to realize automatic detection and precise localization of movements in swimming sports videos. The research aims to solve the problems in motion detection in swimming sports video due to the challenges of fast‐changing movements, large spatial span, and many changes in postures. It seeks to offer technical and theoretical assistance for the future advancement of intelligent sports analysis systems.

There are four parts to the study. The first part analyzes the existing research on human motion detection methods for swimming sports worldwide. The second part describes the proposed human motion detection method based on MS separation STAM for swimming video. In the third part, a large number of experiments are conducted for this method to verify its effectiveness and superiority. The fourth section offers a summary of the study, identifies its short‐term flaws, and suggests fresh lines of inquiry.

## 2. Related Works

Swimming motion video human motion detection can help coaches and athletes accurately analyze the details of movements, optimize technical performance, and improve training and competition effects [12]. In addition, it can also assist in injury prevention and scientific rehabilitation, providing support for the long‐term development of athletes [13]. Therefore, it has been studied by many scholars worldwide. Aiming at improving the efficiency of pose recognition in aerobic sports (e.g., swimming), Liu proposed a convolutional neural network (CNN)‐long and short‐term memory (LSTM) network recognition model by using a combination of CNN and LSTM networks. The study achieved higher recognition accuracy and robustness [14]. Aiming at the problem of insufficient robustness of the graph‐structured swimmer pose estimation method, Cao and Yan [15] proposed a human swimming key point detection model by combining a multidimensional convolutional network and a high‐resolution network. The study achieved higher pose estimation accuracy [15]. In response to the difficulty of existing swimming action video analysis techniques to resist the effects of bubbles, splashes, and light reflections, Giulietti et al. [16] proposed a novel markerless 2D swimmer pose estimation method by combining wearable sensors and swimmer Net networks, thus improving the accuracy of athlete pose recognition. To address the need of assisting divers to accomplish underwater tasks, Liu et al. [17] designed a swimming pose recognition method using a 3D CNN with an underwater dataset and target tracking technique, thus significantly improving the accuracy of pose recognition. Aiming at the effectiveness of machine learning and deep learning techniques in swimming activity recognition, Chen and Hu [18] combined reinforcement learning and inertial measurement units to propose a novel human swimming pose recognition model, thus improving the balance accuracy of pose recognition.

In addition, for the existing underwater swimming pose recognition technology limited by visible light wavelength, Wang et al. [19] proposed a novel recognition method by using light detection and ranging lidar data, combined with a radius outlier removal method and PointNet network, thus overcoming the limitation of light wavelength. To address the need of human pose recognition for enhancing underwater snorkeling, Abdul Rahman et al. [20] proposed a real‐time monitoring model by combining a 3D CNN. The method achieved highly accurate pose recognition by dynamically analyzing the pose of the snorkeler [20]. Aiming at the effect of changing lighting conditions and image quality degradation on the accuracy of swimming pose detection by CNNs, Xu [21] proposed a novel swimming pose recognition model by preprocessing and enhancing the input image, thus maintaining good performance in different environments. Aiming at improving the detection accuracy of intelligent underwater gesture recognition sensors, Fan et al. [22] combined a three‐dimensional CNN with a capacitive stretch sensor to construct a novel swimming gesture recognition model, thus ensuring the accuracy and effectiveness of gesture recognition. Aiming at the time‐consuming and laborious problem of traditional swimming gesture analysis methods, Zhang et al. [23] combined a two‐dimensional CNN with a support vector machine algorithm to propose a diving gesture communication recognition model, thus significantly improving the recognition effect.

In summary, recent studies have made progress in swimming motion video human motion detection, with improvements in recognition accuracy and robustness achieved through CNNs, LSTM networks, multidimensional convolutional networks, and high‐resolution networks. Wearable sensors, LiDAR, and other technologies have also been applied to enhance detection in complex underwater environments. However, existing methods still lack stability when dealing with backgrounds such as air bubbles and light reflections, and most rely solely on either spatial or temporal features, without effectively integrating spatio‐temporal features (STFs), making it difficult to accurately capture MS action details. To address these gaps, this study proposes a method based on MS separation STAM, which improves detection accuracy and adaptability in complex environments, providing new technical support for intelligent analysis and training optimization in swimming.

## 3. Methods and Materials

This section describes the human motion detection method for swimming motion video proposed in the research. The method’s network structure (NS) is presented initially, and then each component of the NS is thoroughly explained. Finally, the final flow of the swimming motion video human motion detection method is summarized.

### 3.1. Human Motion Detection Network Design and Input Processing

**Figure 1:**
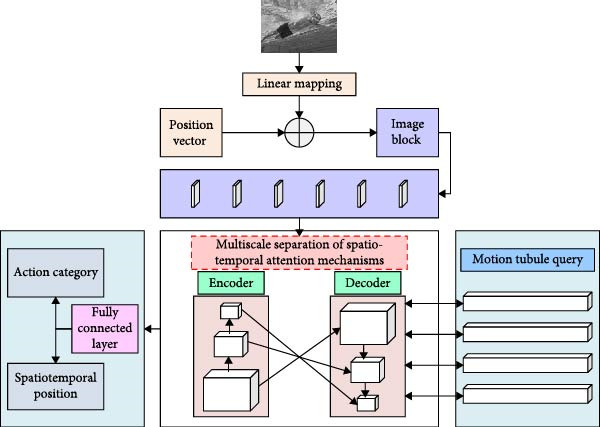
Network structure diagram of the detection method.

In sports video analysis, spatio‐temporal information is crucial, which indicates the changes of objects or human bodies in the video in both spatial and temporal dimensions [24]. The movements in swimming are not only accompanied by the movement of the athlete in spatial location but also show different rhythms and speed changes in time [25]. Existing motion detection methods for sports videos often struggle to capture these complex changes simultaneously in the spatio‐temporal dimension [26]. Some methods may only be able to capture a certain moment of the action, fail to fully track the continuity of the action, or fail to accurately recognize the detailed changes of the action in different spatial locations [27]. Therefore, to address the complexity of swimming motion, the study proposes a human motion detection method for swimming motion video based on MS separation STAM. The method aims to effectively improve the detection ability of complex movements of swimming sports through STAM and MS feature extraction. Figure 1 depicts the method’s overall network topology.

**Figure 2:**
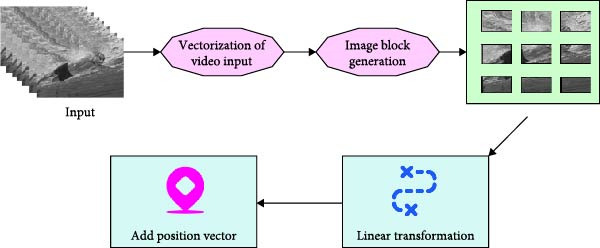
Workflow of image generation module.

As shown in Figure 1, the proposed method consists of four main components: image patch generation, MS separation STAM, motion tube query, and fully connected layers. Video frames are divided into patches and encoded with positional information, then processed by the MS separation STAM to extract temporal and spatial features while adapting to actions of varying speeds and scales. The motion tube query subsequently generates the athlete’s spatio‐temporal trajectory, and the fully connected layers output the action category along with its spatial location and temporal range. On this basis, the study describes each module separately. The specific flow of the image block generation module is shown in Figure 2.

In Figure 2, the workflow of the image generation module is divided into four major steps: vectorization of video input, image block generation, linear transformation, and adding position vectors. It is assumed that the input video clip is denoted as $X\in R^{T\times H\times W\times C}$. Among them, *T* denotes the number of video frames, that is, the length in the time dimension. *H* and *W* denote the height and width of each frame, respectively. *C* is the color channel. Each frame is equally divided into image blocks of size *S* × *S*, and each frame is divided into a total of *N* blocks. The expression for *N* is shown in Equation (1).

(1) N=H×WS2.

Further, each image block is expanded into a one‐dimensional vector and denoted as *x*_*t*,*s*_. Among them, *t* = 1, 2, ⋯, *T* denotes the *t*‐th frame and *s* = 1, 2, ⋯, *S* denotes the first *s* image block. The length *L* of the image block is shown in Equation (2).

(2) L=S×S×C.

Each image block *x*_*t*,*s*_ is mapped to a fixed dimension *d* by linear transformation to obtain a feature representation, as shown in Equation (3).

(3) zt,s=W⋅xt,s+b.

In Equation (3), *z*_*t*,*s*_ denotes the feature representation after linear transformation. *W* denotes the weight matrix used to map the image block to a fixed dimension. *b* denotes the bias term. To preserve the spatio‐temporal location information of each image block, the study further adds spatial location vectors and temporal location vectors to the feature *z*_*t*,*s*_. The final input of each image block is shown in Equation (4).

(4) ht,s=zt,s+ps+qt.

In Equation (4), the spatial position vector *p*_*s*_ is the spatial position of the image block in the frame. The temporal position vector *q*_*t*_ represents the position of the image block in different time frames. *h*_*t*,*s*_ is the feature representation that finally contains the spatio‐temporal position information. By the above steps, the image block generation module transforms the input video clip into a vector representation containing spatio‐temporal position information for processing in the subsequent network.

### 3.2. MS Separation STAM

**Figure 3:**
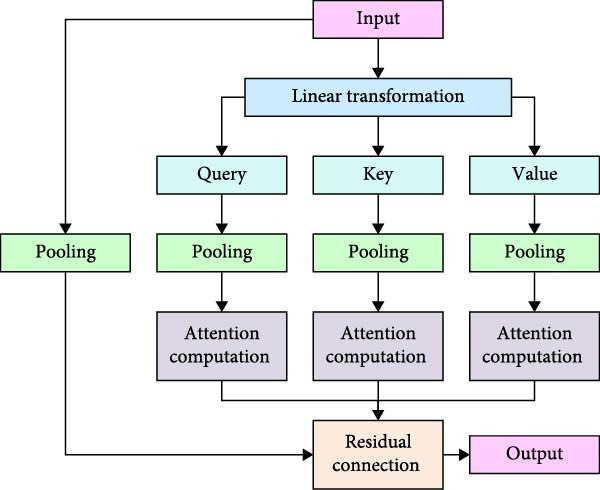
Schematic diagram of the spatial pooling attention module.

After acquiring the video image data with vector representation containing spatio‐temporal location information through the image block generation module, the study proposes an MS separation STAM. It extracts features in both temporal and spatial dimensions separately to enhance the model’s ability to detect complex actions in swimming sports. Based on the MS STF encoder and decoder, the mechanism gradually extracts STFs at different scales by introducing a separation spatio‐temporal pooling attention module (AM), which reduces the amount of computation and preserves the global information. The mechanism consists of two core modules, namely, spatial pooling AM and temporal pooling AM. Among them, the spatial pooling AM aims to extract spatial features at different locations within each image frame, which is schematically shown in Figure 3.

In Figure 3, the spatial pooling AM calculates the attention weights between image blocks mainly within each frame. The downsampling of features is achieved by a pooling operation, which in turn extracts MS spatial features. The input vectors of each spatial location are mapped into queries, keys, and values by linear transformation. The pooling operation is performed before computing the attention to reduce the computation. Subsequently, the pooled attention results are concatenated with the original input residuals to ensure that the output remains spatially consistent. It is assumed that the input feature be $X\in R^{L\times D}$. Among them, *L* = *T* × *H* × *W*, *D* are the feature dimensions of each image block. The length of the feature vector of each image block is obtained after the image block generation module. For each frame, take all the image blocks of that frame, denoted as $X_{t}\in R^{H\times W\times D}$. *X*_*t*_ is first linearly transformed to generate the query, key, and value matrices *Q*_*t*_, *K*_*t*_, and *V*_*t*_, as shown in Equation (5).

(5) Qt=WQ⋅LNXt+psKt=Wk⋅LNXt+psVt=Wv⋅LNXt+ps.

In Equation (5), *W*_*Q*_, *W*_*k*_, and *W*_*v*_ denote the learnable weight matrices, which are used to generate feature vectors for queries, keys, and values, respectively. LN denotes layer normalization, which helps to increase feature stability. *p*_*s*_ denotes the spatial position vector used to retain the positional information of the image block in the frame. To reduce the computational complexity, pooling operations are performed on *Q*_*t*_, *K*_*t*_, and *V*_*t*_. The spatial attention is computed on the query, key, and value after pooling is performed, as shown in Equation (6).

(6) AttentionQtpool,Ktpool,Vtpool=softmaxQtpool⋅KtpoolTD⋅Vtpool.

In Equation (6), $Q_{t}^{\text{pool}}$, $K_{t}^{\text{pool}}$, and $V_{t}^{\text{pool}}$ denote the pooled query, key, and value, respectively; softmax denotes the softmax function that normalizes the dot product results of the query and key to generate the attention weights. $\sqrt{D}$ denotes the scaling factor that prevents the attention value from being too large and affecting the training [28]. To preserve the original spatial feature information, the study connects the attention output to the original input with residuals. On the pooled output, the same pooling operation is performed on the input and summed to ensure consistent dimensionality, as shown in Equation (7).

(7) Yt=AttentionQtpool,Ktpool,Vtpool+PoolLNXt+ps;P.

In Equation (7), $Y_{t}\in R^{H\prime \times W\prime \times D}$ denotes the output of spatial pooling AM. Among them, *H*′ and *W*′ denote the spatial dimensions after pooling. Pool(LN(*X*_*t*_ + *p*_*s*_); *P*) denotes the same pooling operation on the original input features [29, 30]. With the above steps, the spatial pooling AM is able to effectively extract MS spatial features at different locations within each image frame. It retains rich spatial information while reducing computational effort, providing efficient spatial feature input for the subsequent temporal pooling AM.

The temporal pooling AM is used to extract the feature variations of each spatial location in the temporal dimension to capture the dynamic information of the action, and its workflow is similar to that of the spatial pooling AM. First, a linear transformation is performed, and temporal location vectors are added; then, temporal pooling of queries, keys, and values is performed to reduce the computational effort. Finally, the output containing temporal dynamic features is generated by residual concatenation to enhance the continuity understanding of action detection. Overall, the spatial pooling AM extracts MS spatial features within each image frame, while the temporal pooling AM captures the dynamics of the action in the temporal dimension. The two together can enhance the comprehensive detection of complex swimming actions.

### 3.3. MS STF Encoder and Decoder

**Figure 4:**
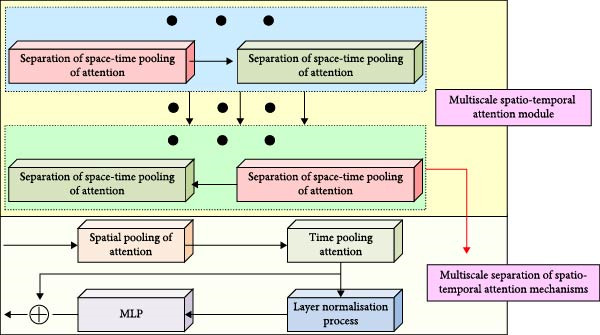
Structure of multiscale spatio‐temporal feature encoder.

After MS separation, STAM is used to extract the STFs of the swimming video; the study further adds an MS STF encoder with decoder to realize accurate action detection. The MS STF encoder consists of multiple MS spatio‐temporal AMs (MSATAMs) stacked together. Each module contains a series of separated spatio‐temporal pooling AMs utilizing MS separation STAM for stepwise extraction of STFs of the video at different scales. The encoder focuses on extracting more detailed local STFs at the initial level, and gradually shifts to abstract global features at deeper levels, thus constructing an MS feature representation. Its structure is shown in Figure 4.

**Figure 5:**
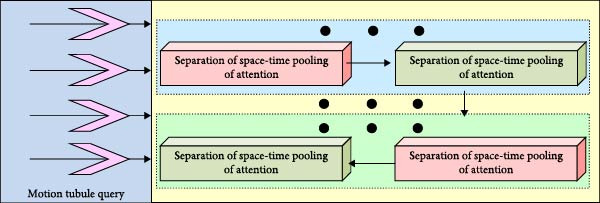
Multiscale spatio‐temporal feature decoder structure.

In Figure 4, in the encoder, each MSATAM forms a feature extraction mechanism from fine‐grained to coarse‐grained by stacking layer by layer on top of the spatial and temporal pooled attention mentioned before. The shallow MSATAM of the encoder usually contains more separated spatio‐temporal pooled attention units to extract localized and fine‐grained action details. In contrast, in the deeper modules, the encoder gradually reduces the number of MSATAMs to simplify the computation and capture global features in the video. Notably, the output features of the encoder not only contain the spatial information of each frame but also integrate the dynamic changes in the temporal dimension, which can reflect the continuity and rhythm of the athlete’s movements in the video more comprehensively. Figure 5 depicts the MS STF decoder’s construction.

In Figure 5, the MS STF decoder has a similar structural design to the encoder, which also consists of a series of MSATAMs. However, its main purpose is to reduce the abstract high‐level features into more concrete and detailed features, thus supporting more accurate motion detection. The decoder also contains an important constituent module, the motion tubule query. Its role is to provide a query vector containing spatio‐temporal information for the target motion to be detected in the video, which is used to accurately localize the region and time of occurrence of the motion. It gradually refines the STFs of the query by interacting with the encoder features layer by layer in the decoder. It is assumed that the query vector of the action tubules in the input video clip is *Q*_tubule_, which contains the initial spatio‐temporal information of the athlete’s actions in the video. *Q*_tubule_ first generates the initial STF representation in the decoder, as shown in Equation (8).

(8) Qtubulel=fSSTPAQtubule,Zencl.

In Equation (8), *f*_SSTPA_ denotes the operation of MSATAM. $Q_{\text{tubule}}^{\left(\text{l}\right)}$ denotes the feature output of the MSATAM at layer *l*. $Z_{\text{enc}}^{\left(\text{l}\right)}$ denotes the feature output of the encoder at layer *l* for fusion with the motion tubule query. After each layer of MSATAM output, the updated features of the motion tubule query are fused with the features of the previous layer of the decoder across the layers to gradually restore more concrete detail information. The fused STF representation is shown in Equation (9).

(9) Qtubulel+1=AttentionQtubulel,Zencl+1.

**Figure 6:**
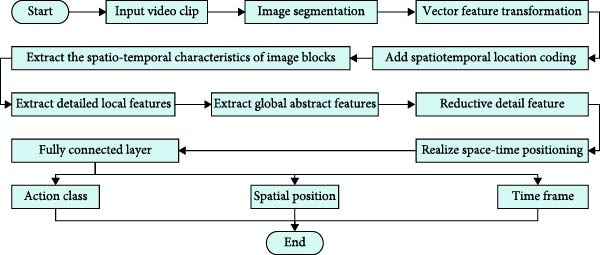
Final flow of human motion detection method in swimming video.

In Equation (9), $Q_{\text{tubule}}^{\left(\text{l}+1\right)}$ denotes the updated query features after the MSATAM at layer *l* + 1. $Z_{\text{enc}}^{\left(\text{l}+1\right)}$ denotes the encoder’s feature output at layer *l* + 1. The motion tubule query continuously refines the spatio‐temporal information in each layer so that it gradually obtains a precise description of the location, details, and categories of the actions. Ultimately, an accurate STF representation is provided at the output of the decoder for the motion detection task. Combining all of the above, the final flow of the research proposed human motion detection method for swimming motion video is shown in Figure 6.

In Figure 6, the final flow of the research proposed method is as follows. The input video clip is first divided into multiple image blocks, and spatio‐temporal location coding is added. Then features are extracted in spatial and temporal dimensions by MS separation STAM, respectively. The encoder extracts STFs layer‐by‐layer from fine‐grained to coarse‐grained. The decoder reduces the feature details step by step through the motion tubule query module to realize the accurate spatio‐temporal localization of the action. Finally, the categories, spatial locations, and temporal ranges of the actions are outputted through the FCL to comprehensively detect and recognize the complex actions in swimming sports videos.

## 4. Results

The study carries out numerous experiments on the suggested detection approach in an attempt to confirm its superiority and validity. First, the MS separation STAM in the method is verified and analyzed in detail. Subsequently, the performance of the whole detection method is validated and analyzed.

### 4.1. Performance Validation of MS Separation STAM

The investigation starts by setting up the experimental environment and determining the required parameters before the experiment begins. Table 1 presents the information.

**Table 1:** Experimental environment configuration and parameter settings.

Hardware environment		Necessary parameter settings	
CPU	Intel Core i7‐11700K	Resolution	1920 × 1080
GPU	NVIDIA RTX 3080	Frame rate	60 fps
Memory	32 GB RAM	Encoding format	H.264
Storage	1 TB SSD	Batch size	32
Camera device	1080p HD camera, supports 30 fps or 60 fps recording	Learning rate	0.001
Tripod	For stabilizing the camera	Optimizer	Adam
Underwater camera	Waterproof camera	Training epochs	100
Software environment		Image block size	64 × 64
Operating system	Windows 10	Data augmentation	Random cropping, rotation, and flipping
Deep learning framework	TensorFlow 2.x	—	—
Development environment	Jupyter notebook	—	—
Video processing tool	OpenCV	—	—
Data annotation tool	LabelImg	—	—

Based on Table 1, the SwimNet dataset and the UCF Sports dataset were selected as the data sources for the experiments. The SwimNet dataset focuses on capturing the movements of swimmers in different strokes, containing multiple training and competition videos with frame‐by‐frame annotations covering key body parts and action phases [31]. The annotations were performed by three annotators with a professional background in swimming technique analysis, following a unified annotation protocol and using the LabelImg tool. To ensure quality, ~15% of the samples were cross‐labeled, achieving a Cohen’s kappa coefficient of 0.91, indicating excellent interannotator consistency. The UCF Sports dataset contains videos of various sports, including multiple swimming actions, and its official annotations were directly adopted as supplementary data [32]. Both datasets were split into training and testing sets at a 7:3 ratio, and the splitting rules and preprocessing scripts will be released together with the source code to ensure experimental reproducibility. The study first evaluates the performance of MS separation STAM in feature extraction and selects the single‐scale STF extraction mechanism as a comparison method. The results are shown in Figure 7.

Figure 7Comparative results of feature extraction performance. (a) Comparison of feature mapping accuracy. (b) Comparison of feature importance.

**Figure:**
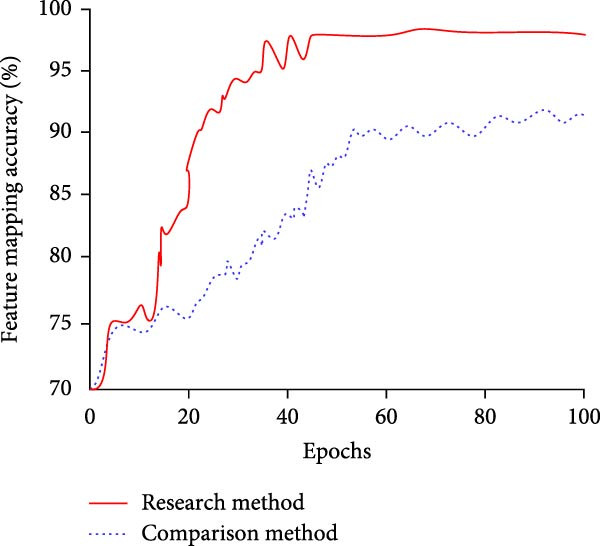
(a)

**Figure:**
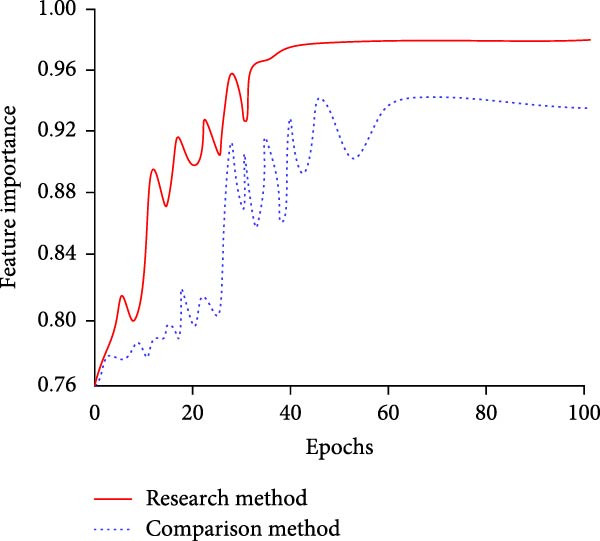
(b)

In Figure 7a, the feature mapping accuracy of the MS separation STAM increases rapidly with the number of training rounds. Its convergence is completed at the 43rd iteration, and the final accuracy reaches 97.34%. While the accuracy of the single‐scale STF extraction mechanism still fluctuates at 80–100 iterations and only maintains at about 90%. In Figure 7b, the MS separation STAM basically completes convergence after the 40th iteration. At this time, the feature importance reaches 0.982, while the single‐scale STF extraction mechanism completes the convergence after 65 iterations, and the importance is only 0.938 at the time of convergence. It can be concluded that the MS separation STAM not only accelerates the convergence of the model but also can extract more discriminative and stable features. On this basis, the study evaluates the contribution of the STAM to the model recognition accuracy and compares the detection methods with and without the STAM. Table 2 displays the findings.

**Table 2:** Influence of spatio‐temporal attention mechanism on recognition accuracy.

Model	Action type	Accuracy (%)	Recall (%)	F1 score (%)
With a spatio‐temporal attention mechanism	Freestyle	94.52	93.67	94.09
	Breaststroke	95.23	94.78	95.00
	Backstroke	93.87	92.32	93.09
	Butterfly	92.46	91.58	92.02

Without a spatio‐temporal attention mechanism	Freestyle	89.34	88.67	89.00
	Breaststroke	90.23	89.12	89.67
	Backstroke	88.56	87.45	88.00
	Butterfly	87.34	86.78	87.06

In Table 2, the model with the STAM significantly outperforms the model without the attention mechanism in terms of recognition accuracy, recall, and F1 score, which reflects the advantages of this mechanism in swimming action detection. Specifically, the average values of accuracy, recall, and F1 score of the model adopting the attention mechanism reach 94.02%, 93.09%, and 93.56% on all types of actions, respectively, whereas the corresponding metrics of the model without the attention mechanism are only 88.87%, 88.01%, and 88.44%. This indicates that the STAM can capture the action features more accurately and comprehensively. It can effectively improve the detection accuracy and comprehensiveness of the model, which is suitable for intelligent detection of complex actions in swimming sports videos. Further, the study evaluates the performance of MS separation STAM in terms of computational complexity. The results are shown in Figure 8.

Figure 8Computational complexity analysis. (a) Processing time per frame. (b) Memory usage. (c) CPU usage. (d) GPU usage.

**Figure:**
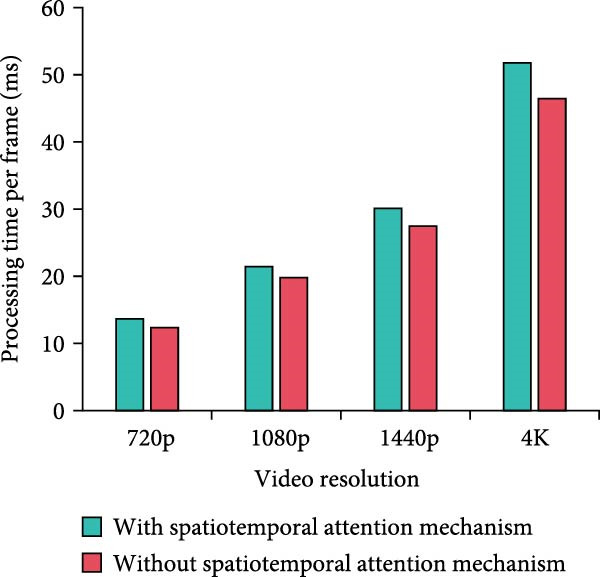
(a)

**Figure:**
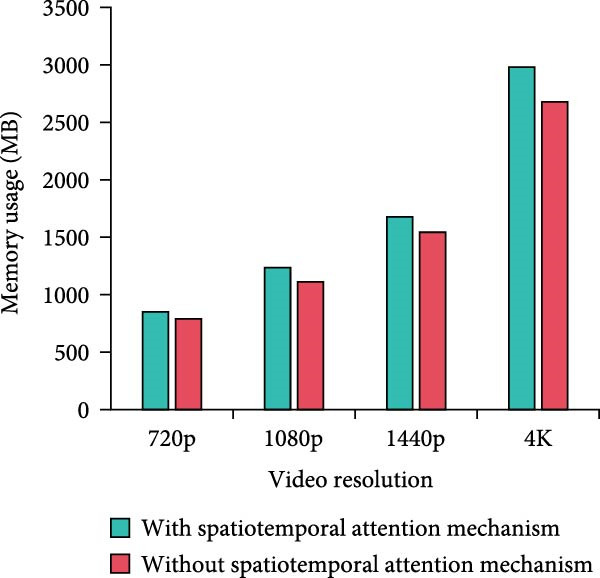
(b)

**Figure:**
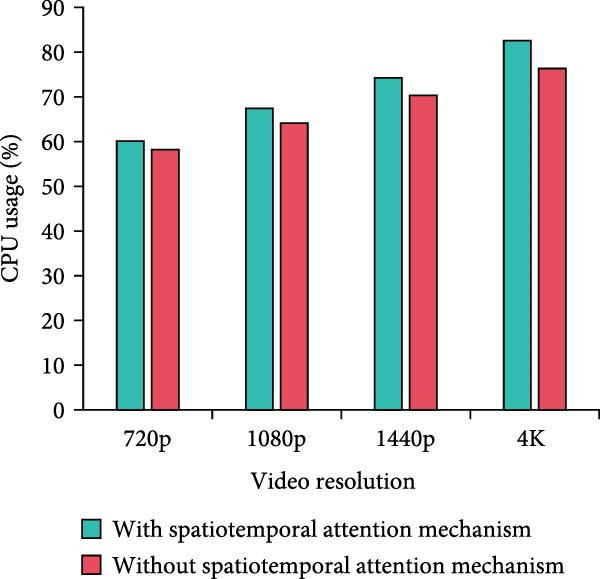
(c)

**Figure:**
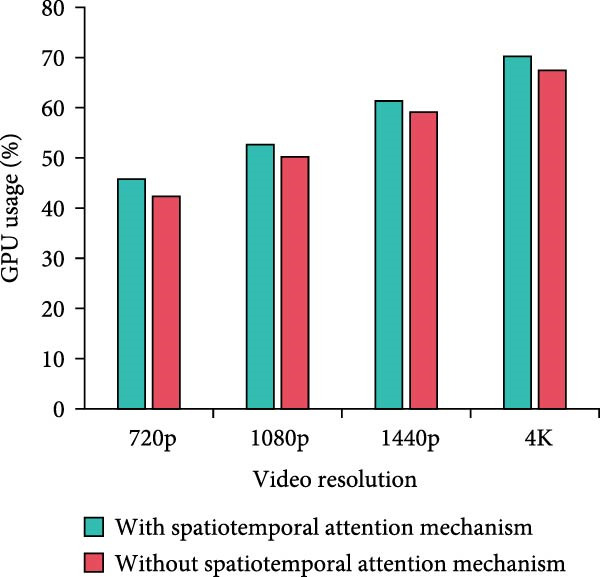
(d)

In Figure 8a, the per‐frame processing time of the method with the MS separation STAM increases by a small amount of time over the method without the mechanism at all resolutions. At 4K resolution, the increase is 11.5% from 46.45 to 51.78 ms. Overall, it stays within the acceptable range. In Figure 8b, the memory requirements of the MS mechanism are slightly higher, but the increase is not significant. For example, it increases from 1112.45 to 1234.89 MB at 1080p resolution, which is an increase of 11%. The increase in memory requirement is kept at a low level. In Figure 8c,d, the model using MS separation STAM shows a small increase in CPU and GPU occupancy. For example, at 4K resolution, the CPU utilization increases from 76.34% to 82.56%, which is around 8%. GPU utilization increases from 67.45% to 70.23%, an increase of only 4.1%. The model with MS separation STAM does increase in computational complexity. However, such a computational cost is reasonable and valuable considering the improvement in detection accuracy brought about by the mechanism. The increase in resource consumption of the MS mechanism is more pronounced at high resolution. Therefore, the mechanism can play a better role in scenarios with higher performance requirements, but the allocation of hardware resources also needs to be considered.

To further validate the effectiveness of the proposed MS separation STAM, a small‐scale additional ablation study was conducted in which the MS STAM was replaced with two representative AMs: a standard transformer‐based spatio‐temporal AM and a 3D‐CNN‐based nonlocal AM. All other experimental settings were kept consistent. The results are shown in Table 3.

**Table 3:** Additional ablation comparing MS separation STAM with representative attention modules.

Attention module	Accuracy (%)	Recall (%)	F1 score (%)	Inference time (ms/frame)
MS separation STAM	94.02	93.09	93.56	51.78
Transformer‐based	91.45	90.12	90.78	66.34
3D‐CNN nonlocal	92.03	91.01	91.51	53.89

From Table 3, it can be seen that the MS separation STAM achieves an accuracy of 94.02% and a recall of 93.09%, both higher than the 91.45% and 90.12% of the Transformer‐based module and the 92.03% and 91.01% of the 3D‐CNN Non‐local module. In addition, benefiting from the introduction of pooling in its design, the MS separation STAM achieves an inference time of 51.78 ms per frame, which is significantly faster than the 66.34 ms per frame of the transformer‐based module, while remaining close to the 53.89 ms per frame of the 3D‐CNN module. This advantage stems from differences in structural design: unlike standard transformer‐based video models that compute global attention jointly over the spatio‐temporal dimension, the MS separation STAM first decouples spatial and temporal attention, extracting MS features separately through spatial pooling and temporal pooling modules, and then fusing them to capture local dynamic changes while preserving global context. Furthermore, pooling is applied before attention computation to reduce the number of tokens, thereby maintaining accuracy while lowering computational complexity.

Finally, the study conducts an adaptation test for different movement variations. A total of four groups are designed, which are fast movement speed+large amplitude of movement (AOM), fast movement speed + small AOM, slow movement speed+large AOM, and slow movement speed + small AOM, which are named as G1–G4, respectively. The results are shown in Figure 9.

Figure 9Adaptability test results of different motion changes. (a) Freestyle. (b) Breaststroke. (c) Backstroke. (d) Butterfly.

**Figure:**
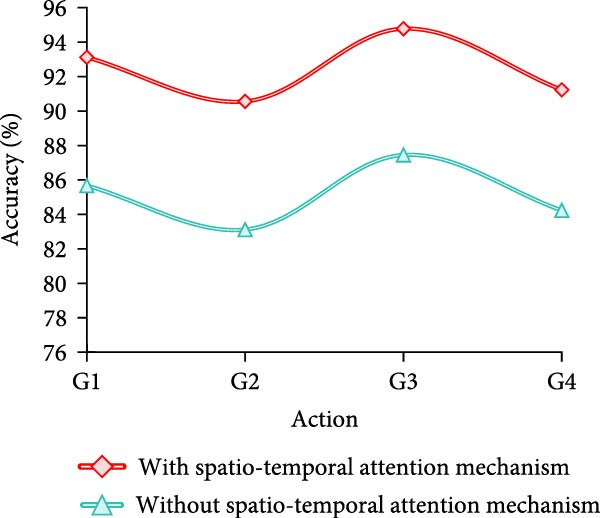
(a)

**Figure:**
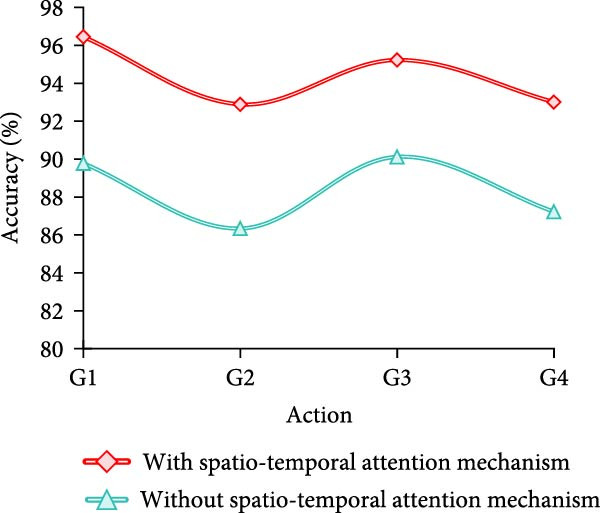
(b)

**Figure:**
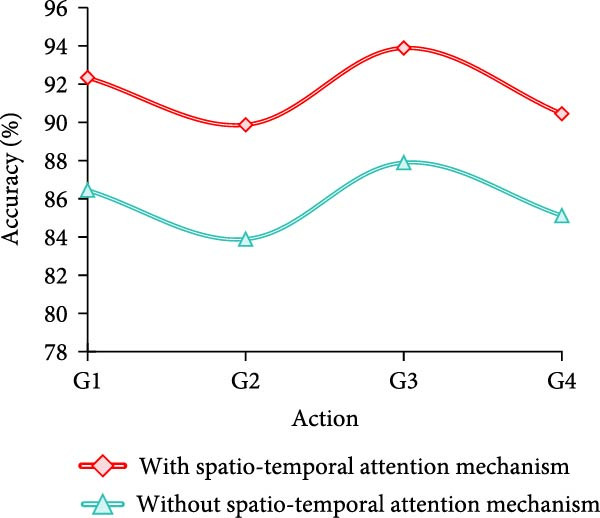
(c)

**Figure:**
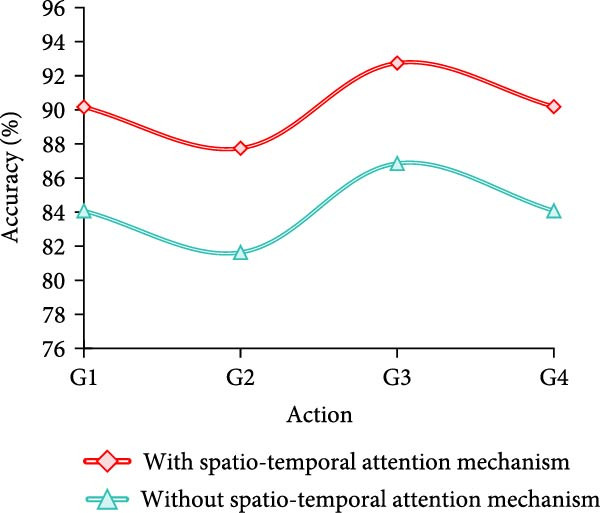
(d)

Combining the results in Figure 9a–d, it can be concluded that the model of MS separation STAM has a high and stable detection accuracy under different swimming motions, speeds, and amplitude variations, which is generally maintained above 89%. In particular, the accuracy is highest in slow and large amplitude movements, reaching more than 95%. On the other hand, the accuracy of the models that do not use this mechanism fluctuates greatly, especially in fast, small‐amplitude movements, where the accuracy decreases significantly. For example, backstroke and butterfly are only 83.89% and 83.23%, respectively. Overall, the MS separation STAM improves the model’s adaptability and detection accuracy under complex movement changes.

### 4.2. Performance Validation of Human Motion Detection Method for Swimming Motion Video

To further validate the performance advantages of the proposed method, the study first chooses the more advanced inflated 3D ConvNet (I3D) and the SlowFast network as comparison methods. The former captures the STFs of the video by extending the 2D convolution to 3D convolution, while the latter captures the features of different motion speeds in the video by parallelizing the slow and fast paths. The test results in the training set are shown in Figure 10.

Figure 10Results of different methods in the training set. (a) Comparison of the recall rate. (b) Comparison of false detection rate.

**Figure:**
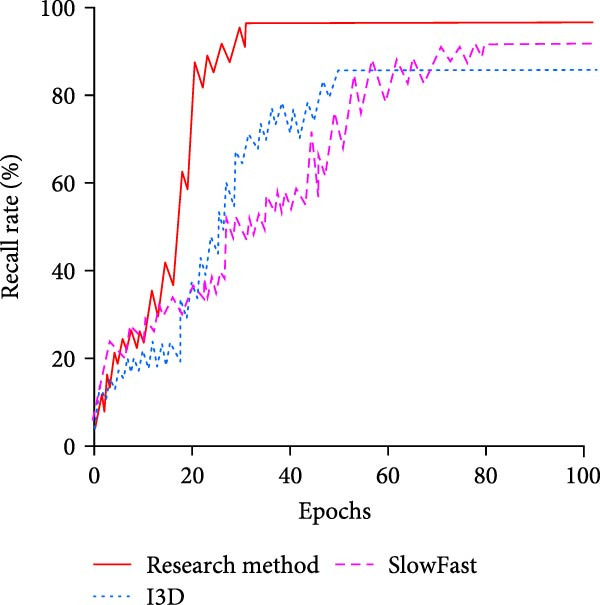
(a)

**Figure:**
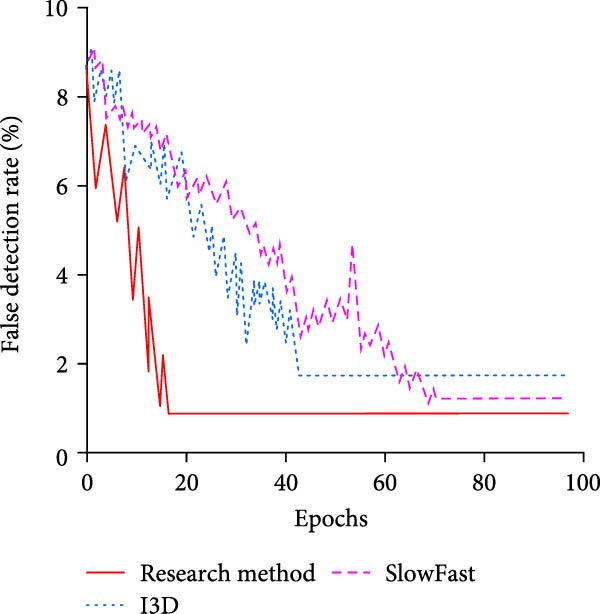
(b)

In Figure 10a, the recall of the research proposed method, I3D, and the SlowFast network increases with the iterations. The iterations at convergence are 30, 50, and 75, respectively. The recall at convergence is 96.48%, 83.12%, and 90.37%, respectively. In comparison, the proposed method of the study has faster convergence with higher recall. In Figure 10b, the research‐proposed method achieves convergence at the early stage of training, that is, after less than 20 iterations, when the false alarm rate is only 0.86%. I3D completes convergence after 42 iterations, and the false alarm rate at convergence is 1.94%. SlowFast network achieves convergence after 70 iterations, and the false alarm rate at convergence is 1.28%. Overall, the optimization of the model structure and feature extraction mechanism of the proposed methods in the study makes them more suitable for the complex features of swimming motion videos, and thus they perform well in terms of recall and false alarm rate. To evaluate the performance of different methods in complex underwater environments in more detail, the study divides the scene into three different interference conditions, namely bubble interference, light reflection interference, and multiple interference (bubble and light reflection at the same time). It is compared with I3D and SlowFast networks. Table 4 displays the findings.

**Table 4:** Test results of anti‐interference in water.

Method	Normal scene accuracy (%)	Bubble interference accuracy (%)	Light reflection interference accuracy (%)	Multiple interference accuracy (%)
SwimNet data set				
Research method	94.56	90.23	88.67	86.34
I3D	91.23	84.56	82.78	80.12
SlowFast	92.45	86.34	84.45	82.23
UCF Sports dataset				
Research method	93.78	89.45	87.23	85.01
I3D	90.12	83.23	81.56	78.45
SlowFast	91.34	85.12	83.67	80.89

In Table 4, on both the SwimNet and UCFSports datasets, the accuracy of the research method is higher than that of I3D and SlowFast under all interference conditions, which shows stronger robustness. On the SwimNet dataset, the accuracy of the research method is 94.56% in normal scenes and remains at 86.34% under multiple disturbances. It outperforms I3D and SlowFast, especially under light reflection and multiple interference. In the UCF Sports dataset, the accuracy of the research method is slightly lower overall, but still better than the other methods under complex interference conditions. Especially under multiple disturbances, its accuracy is 85.01%, showing better adaptability and stability. On the whole, the proposed method performs well in complex underwater environments and is more suitable for dealing with multiple interference scenarios. Further, the study confirms that the suggested approach works well in real‐time video situations. The results are shown in Figure 11.

Figure 11Real‐time video detection results. (a) Comparison of the processing frame. (b) Comparison of the accuracy rate.

**Figure:**
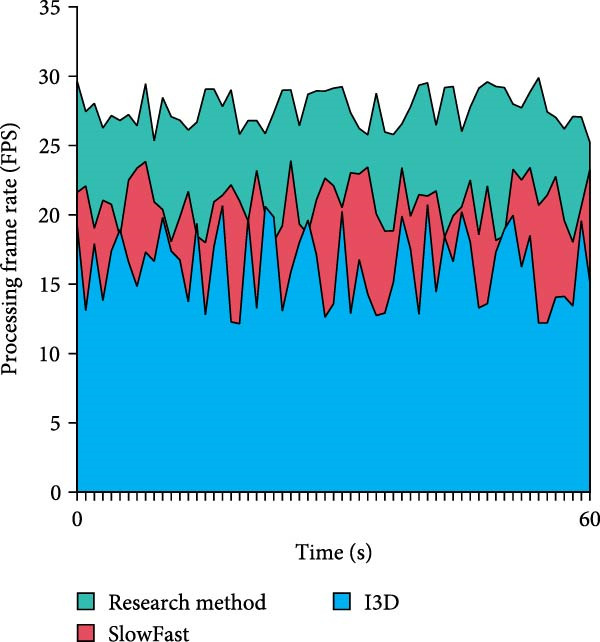
(a)

**Figure:**
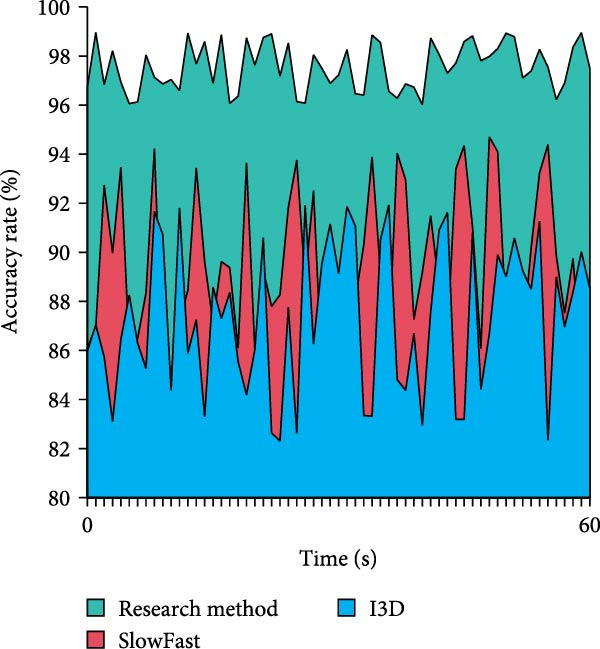
(b)

In Figure 11a, the processing frame rate of the research proposed method is basically stabilized between 25 and 30 FPS throughout the 60‐s real‐time video test. It exhibits high real‐time processing efficiency with less fluctuation. The SlowFast method’s frame rate, which varies more than the study method’s, ranges between 15 and 25 FPS. The I3D method has the lowest frame rate, which basically stays around 15FPS. In Figure 11b, the research‐proposed method maintains a high accuracy in real‐time video detection, with a fluctuation interval of 96%–99% and a similarly small fluctuation range. While the accuracy of the SlowFast method fluctuates between 85% and 95%, the accuracy of the I3D method fluctuates between 82% and 92%. Both have a large fluctuation range and low overall accuracy. This displays that the research proposed method has higher processing efficiency and more stable detection accuracy in real‐time video detection. Furthermore, to evaluate the real‐world deployability of the proposed method on resource‐constrained devices, an additional test was conducted on the NVIDIA Jetson Xavier NX edge device (8 GB memory, 384 CUDA cores, 48 Tensor cores). The experimental settings were kept consistent with the previous tests, and the resolution was set to 1080p. The results are shown in Table 5.

**Table 5:** Edge device benchmark results on NVIDIA Jetson Xavier NX.

Method	Accuracy (%)	FPS	Memory usage (MB)	GPU utilization (%)
Research method	93.87	18.6	2105	72.4
I3D	90.12	16.4	2256	75.8
SlowFast	91.34	17.2	2189	73.6

As shown in Table 5, the research method achieves an accuracy of 93.87% on the Jetson Xavier NX, higher than the 90.12% of I3D and the 91.34% of SlowFast, demonstrating the best detection accuracy. In terms of frame rate, the research method reaches 18.6 FPS, slightly higher than SlowFast’s 17.2 FPS and significantly higher than I3D’s 16.4 FPS, with all three meeting near real‐time detection requirements. For memory usage, the research method requires 2105 MB, which is lower than the 2256 MB of I3D and the 2189 MB of SlowFast. Regarding GPU utilization, the research method is also relatively lower at 72.4%, compared to 75.8% for I3D and 73.6% for SlowFast. Overall, the research method achieves a better balance between accuracy, processing speed, and resource consumption, making it more suitable for deployment in resource‐constrained embedded systems such as poolside edge devices. Finally, the study selects the human motion detection methods from [16, 18, 21] for the comparison of confusion matrices. Eleven common swimming motions are selected. The results are shown in Figure 12.

Figure 12Confusion matrix comparison results. (a) Confusion matrix of [16]. (b) Confusion matrix of [16]. (c) Confusion matrix of [21]. (d) Confusion matrix of the research.

**Figure:**
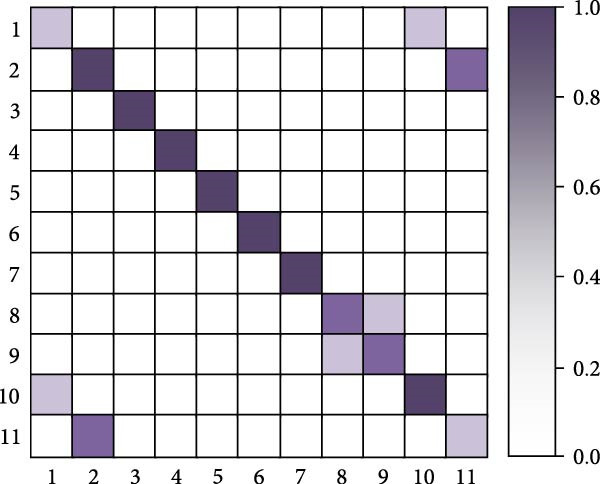
(a)

**Figure:**
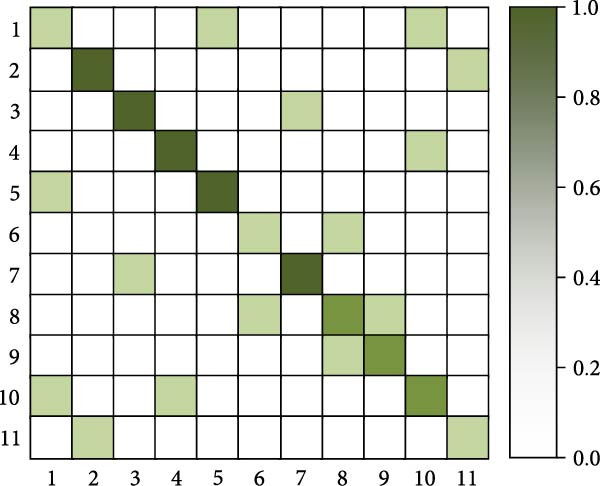
(b)

**Figure:**
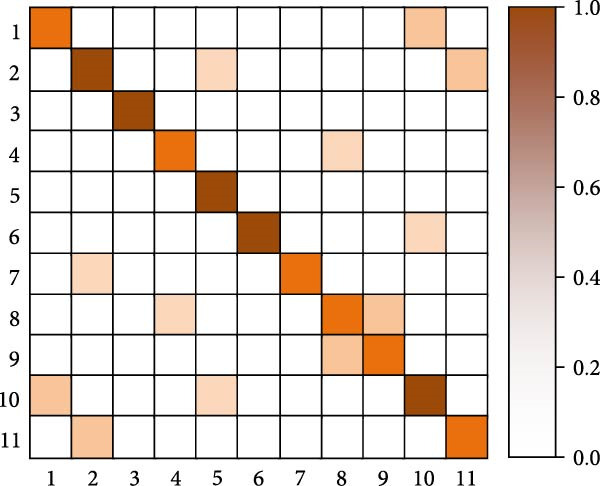
(c)

**Figure:**
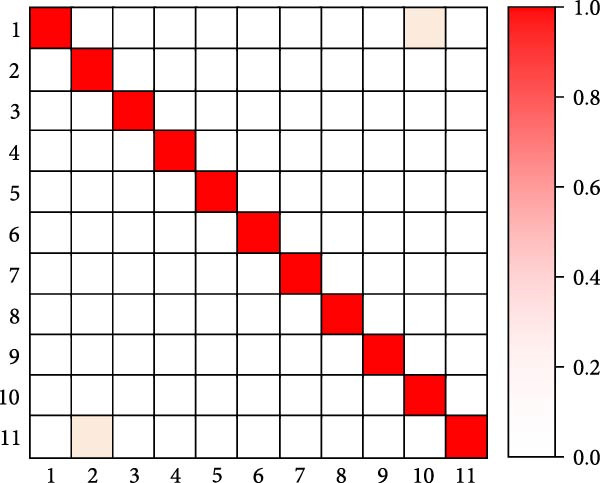
(d)

In Figure 12, 1–11 are represented as freestyle sliding arm, breaststroke sliding arm, backstroke sliding arm, butterfly sliding arm, freestyle kicking leg, breaststroke kicking leg, backstroke kicking leg, butterfly kicking leg, turn around, underwater leg striking, and departure dive. Combining the results of the four confusion matrices in Figure 12a–d, the human motion detection of the swimming motion video from [12] performs worse. The main diagonal of the confusion matrix is relatively fuzzy, and there are more misclassifications in the off‐diagonal region, and the confusion between behavioral categories is more obvious. In contrast, the main diagonal of [10] is more obvious, indicating that its recognition ability is better than [11, 12]. For the method proposed in the study, it performs better than the method in [10]. Its presence of only two color blocks in the nondiagonal region indicates that the proposed method is well able to achieve human motion detection in complex swimming motion videos. Further analysis of typical attention map patterns and failure cases reveals that, in correctly detected samples, the spatial attention primarily focuses on key regions, such as arm strokes and leg kicks, while the temporal attention accurately captures the start and end points, as well as the rhythm changes of the actions. In failure cases, occlusion by water splashes, light reflections, and overlapping postures of multiple swimmers are identified as the main causes of false detections and missed detections.

## 5. Discussion and Conclusion

Aiming at the problems of rapid motion changes and complex underwater environment interference in human motion detection of swimming motion video, the study proposed a detection method based on MS separation STAM. The method extracted and fused features of different scales in spatial and temporal dimensions through an encoder–decoder architecture, which effectively improved the detection of complex movements in swimming sports. The experimental results indicated that the feature mapping accuracy of the method reached 97.34% after 43 iterations, while the feature importance reached 0.982 after the 40th iteration, which was better than the single‐scale method. In terms of recognition accuracy, the method achieved an average accuracy of 94.02%, and the recall and F1 scores were also significantly better than those of the model without the attention mechanism. In the action change adaptation test, the detection accuracy of the method under different actions, speeds, and amplitude changes generally remained above 89%, and the accuracy in slow and large‐sized actions exceeded 95%. The method performed better in terms of recall and false alarm rate compared to I3D and SlowFast networks. Its accuracy was 94.56% in normal scenes in the SwimNet dataset, and remained 86.34% under multiple interference. The real‐time video detection results indicated that the processing frame rate of the method was stable at 25–30 FPS, and the accuracy fluctuation interval was 96%–99%, showing high real‐time processing efficiency and stability. The study presents an effective method for the detection of human motion, specifically for the analysis of swimming motion videos. However, the enhanced precision of the method also elevates the computational burden. Subsequent research may further refine the model architecture to reduce the demand for computational resources and investigate additional athletic activities to augment the model’s generalizability.

## Conflicts of Interest

The author declares no conflicts of interest.

## Funding

The author received no specific funding for this work.

## Data Availability

All data generated or analyzed during this study are included in this published article.
